# Qualified Nurses’ Perceptions of Cultural Competence and Experiences of Caring for Culturally Diverse Patients: A Qualitative Study in Four European Countries

**DOI:** 10.3390/nursrep12020034

**Published:** 2022-05-05

**Authors:** Isabel Antón-Solanas, Beatriz Rodríguez-Roca, Valérie Vanceulebroeck, Nuran Kömürcü, Indrani Kalkan, Elena Tambo-Lizalde, Isabel Huércanos-Esparza, Antonio Casa Nova, Nadia Hamam-Alcober, Margarida Coelho, Teresa Coelho, Yannic Van Gils, Seda Degirmenci Öz, Arzu Kavala, Ana B. Subirón-Valera

**Affiliations:** 1 Department of Physiatry and Nursing, Faculty of Health Sciences, Universidad de Zaragoza, 50009 Zaragoza, Spain; ianton@unizar.es (I.A.-S.); subiron@unizar.es (A.B.S.-V.); 2 Research Group Nursing Research in Primary Care in Aragón (GENIAPA) (GIIS094), Institute of Research of Aragón, 50009 Zaragoza, Spain; 3 Department of Nursing, AP University of Applied Sciences and Arts, 2000 Antwerpen, Belgium; valerie.vanceulebroeck@ap.be (V.V.); yannic.vangils@ap.be (Y.V.G.); 4 Faculty of Health Sciences, Halit Aydin Campus 38, Istanbul Aydin University, Istanbul 34295, Turkey; nurankomurcu@aydin.edu.tr (N.K.); sedadegirmenci@aydin.edu.tr (S.D.Ö.); arzukavala@aydin.edu.tr (A.K.); 5 Nutrition and Dietetics Department, Istanbul Medipol University, Istanbul 34083, Turkey; indrani.kalkan@medipol.edu.tr; 6 Instituto de Investigación Sanitaria, Hospital Universitario Miguel Servet, 50009 Zaragoza, Spain; etambo@salud.aragon.es; 7 Faculty of Health Sciences, Universidad San Jorge, Autovía Mudéjar, 50830 Zaragoza, Spain; ihuercanos@usj.es; 8 Instituto Politécnico de Portalegre, School of Health Sciences, 7300-110 Portalegre, Portugal; casanova@ipportalegre.pt; 9 Servicio Aragonés de Salud, Hospital Materno Infantil, 50009 Zaragoza, Spain; nhamam@salud.aragon.es; 10 Instituto Politécnico de Portalegre, School of Education and Social Science, 7300-109 Portalegre, Portugal; margco@ipportalegre.pt (M.C.); teresa.coelho@ipportalegre.pt (T.C.); 11 Department of Nursing and Midwifery Sciences, University of Antwerp, 2610 Wilrijk, Belgium; 12 Research Group Safety and Care (GIISA0021), Institute of Research of Aragon, 50009 Zaragoza, Spain; 13 Research Group Water and Environmental Health (B43_20R), University Institute of Research in Environmental Science of Aragón, University of Zaragoza, 50009 Zaragoza, Spain

**Keywords:** cultural competence, cultural diversity, Europe, health equity, health services, multiculturalism, nurses, nursing, qualitative research

## Abstract

Background: European nurses are expected to provide appropriate care for patients from diverse cultural backgrounds. However, there is limited knowledge and understanding of this process. The aim of this study was to analyse the perceptions of culture and experiences of caring for patients from diverse cultural backgrounds of a purposive sample of qualified nurses from four European countries, namely Belgium, Portugal, Spain and Turkey. Methods: A qualitative phenomenological approach was selected in order to understand complex phenomena through the participants’ lived experiences, meanings and perspectives. Individual interviews and focus groups took place with 28 staff nurses and 11 nurse managers from four European countries. The sociodemographic and cultural characteristics of the sample were described and analysed using descriptive statistics. Qualitative data were transcribed verbatim, translated into English and analysed following Braun and Clark’s phases for thematic analysis. Results: Five themes and twelve subthemes emerged from thematic analysis of the transcripts. The themes included: (1) relevance of culture for nursing; (2) culture in the healthcare service; (3) qualities of the healthcare professionals; (4) challenges to culturally competent care; (5) becoming a culturally competent nurse. Conclusions: There are challenges to the delivery of culturally congruent care, namely language and communication difficulties, prejudices and stereotyping in the health service, a tendency for ethnocentrism, a lack of education and training in cultural competence and a lack of support from the health service to facilitate new ways of acting.

## 1. Introduction

According to the European Commission (2014), “social inclusion is at the core of the European Social Model and European values enshrined in the Lisbon Treaty” [1]. Nevertheless, in the past few years, social exclusion and inequality have emerged as a major concern in European society. The project funded by the Erasmus+ program under Key Action 203 Strategic Partnerships for Higher Education was conceived to address this issue. It represents a collaboration between 4 European HEIs. In this paper, we present the results from our investigation of European nurses’ perceptions of culture and experiences of caring for patients from diverse cultural backgrounds.

In the last few decades, European countries have seen some rapid changes in their demographic patterns [2]. Once characterised by their homogenous societies, they have now become multicultural societies striving to adapt to the new state of affairs. The reasons behind this transformation are diverse and vary slightly from one nation to another. For instance, Spain, Turkey and Portugal are geographically in close proximity to the African continent, one of the main sources of immigration to the European Union (EU). Other reasons for the recent increase in the numbers of immigrants and refugees include the steady increases in most EU countries’ national gross domestic product (at least until recently); Spain, Portugal and Belgium’s previous colonial history; and Turkey’s proximity to the Syrian border, where civil war rages on, forcing thousands to leave home and country. These are factors influencing healthcare and highlighting the need for cultural competence among health professionals [3,4].

Research findings have shown that health disparities exist between minority and non-minority groups. Cultural determinants of health such as unequal access to care, poverty, racism, intercultural communication difficulties and ineffective provider–patient interactions have been identified as some of the factors responsible for these disparities [2,5,6,7]. In other words, immigrants, refugees and even native people belonging to cultural minorities, such as Gypsy Roma, are at greater risk of poor health.

In 2018, the European Commission’s Expert Panel on Effective Ways of Investing in Health [8] proposed the following actions to ensure equitable access to healthcare: (1) people should have access to good information about health services in their own language; (2) health services should be culturally sensitive and appropriate to meet the needs of all, including diverse patient populations; (3) healthcare services should be culturally appropriate and acceptable through the provision of interpretation and translation facilities, standards for cultural competence, diversity in the health workforce and adaptation of diagnostic and treatment methods. From a professional ethics point of view, professional mandates compel nurses to seek to provide culturally competent care [9]. At the European level, Sasso et al.’s [10] Code of Ethics and Conduct for European Nurses stands on thirteen principles including “respect for human dignity, human rights and equitable access to care and treatment for everybody”. In addition, it clearly states that “patients have the right to fair and equal access to quality healthcare and treatment from nurses, according to their needs”. It is, therefore, a professional and a moral obligation for nurses to be culturally competent in order to provide high-quality transcultural care to diverse patients [11].

Transcultural nursing involves the study of cultural differences and similarities in health and illness emerging from each cultural group’s underpinning societal and organisational structures [5]. Such an understanding of cultural values, beliefs and practices is the foundation for culturally congruent care; care that meets every patient’s needs, is meaningful to them and supports their lifestyle [12]. Nurses are expected to be able to provide appropriate care for diverse groups, and to ensure that patients’ human rights are respected regardless of (but not limited to) their racial, ethnic, gender, socioeconomic and religious background [6]. However, there is limited knowledge and understanding of this process [5,7,11] in European health services.

The aim of this study is to analyse the perception of culture and experience of caring for patients from diverse cultural backgrounds of a purposive sample of qualified nurses from four European countries, namely Belgium, Portugal, Spain and Turkey.

## 2. Materials and Methods

### 2.1. Design

As the research aimed to elicit the participants’ perceived level of cultural competence and their experience of providing nursing care to a diverse patient population, a qualitative phenomenological approach was selected. This was appropriate, as phenomenological research is a qualitative research method that seeks to understand complex phenomena through the participants’ lived experience, meaning and perspectives [13]. The COREQ reporting guidelines were used in both the framing and reporting of this study to guarantee that sufficient details on the methods of data collection, analysis and interpretation were provided (Table S1).

### 2.2. Participants

The study population consisted of qualified nurses from hospitals and primary care providers in Spain, Belgium, Turkey and Portugal. A purposive sample representing 11 nurse managers (NM) and 28 staff nurses (SN) from a variety of professional backgrounds and areas of expertise was recruited.

In order to limit the possibility of selection bias arising from the purposive sampling method, we used clearly defined selection criteria in order to identify the potential participants. Inclusion criteria for taking part in the study included:

(1) SN and NM employed in a healthcare setting in one of the four participating countries;
(2) Individuals with at least two-years of post-qualifying experience working with patients from diverse cultural backgrounds;
(3) Individuals who agreed to the conditions of the study and gave informed consent to participate.

We excluded from our sample any participants with less than one year’s experience working in their current service.

Access to the sample was gained through previously identified gatekeepers, namely NM or nursing coordinators or directors from each of the study sites. These gatekeepers were contacted directly by the research team via professional email.

### 2.3. Methods of Data Collection

The data were collected from May to December 2020 in the four participating countries. We conducted 5 focus groups comprising 4–6 Turkish, Portuguese and Spanish SN and 4 personal interviews with 4 Belgian SN, either face-to-face or online depending on each country’s circumstances at the time, as well as the participants’ preferences. The original plan was to collect the data through focus groups only. However, due to the circumstances arising from the COVID-19 pandemic in Belgium, a decision was made to carry out personal interviews with the participants instead. In addition, we conducted 11 personal interviews with the NM. The NM did not participate in the focus groups, as participant interaction might have been disrupted if power differentials existed between them and the SN [14]. Instead, this method of data collection was chosen because it can elicit rich, culturally grounded insights into people’s experiences [15].

Both the focus groups and the personal interviews took place in a neutral and safe environment and in the participants’ own language; the sessions were conducted by an academic from each of the study sites, all of whom were experienced in qualitative research. The personal interviews and focus groups ranged from 20 to 60 min in duration. All of the data were audio-recorded and transcribed verbatim. The results were later translated into English by the academic undertaking the interview, all of whom were fluent in English. All the researchers used the same (previously agreed) interview guide developed by the principal investigator (IA-S) and based on the literature review. Focus group questions with SN addressed topics such as how one’s own culture influences practice; whether participants had had formal or experiential training in cultural competence; and how conflict emerging when different cultural values and beliefs clash was dealt with in practice (Table 1).

Interview questions with NM addressed topics such as barriers and facilitators to providing culturally safe nursing care to patients from diverse cultural backgrounds, challenges to cultural competence training, and preparedness of the healthcare service to deliver transcultural care (Table 2).

The participants were also invited to complete a sociodemographic questionnaire with the aim of describing the sample, including the following variables: age (years), gender, marital status, race or ethnicity, religious affiliation, socioeconomic level, country of birth, country of work, professional experience (years), education level, language competence, cultural competence training, involvement with diverse patients or organizations, experience working with patients from diverse backgrounds, and experience living abroad for at least 3 months.

### 2.4. Data Analysis

A descriptive statistics approach was used to analyse the sociodemographic data using frequency and percentage values for qualitative variables and means and standard deviations for quantitative ones.

Anonymised transcripts were analysed qualitatively following Braun and Clark’s [16] phases for thematic analysis, namely familiarising researchers with data, generating initial codes, searching for themes, reviewing themes, defining and naming themes, and producing the report. The scientific quality was guaranteed by using a range of techniques, including:

- Space triangulation was achieved by interviewing NM and SN from different healthcare services to check the consistency of findings over multiple sites;
- An audit trail was kept of the researchers’ decision-making process and critical self-reflection;
- Frequent contact between authors was maintained in order to promote further discussion on emerging themes and potential biases.

Two researchers (ABS-V; BR-R) manually analysed the transcripts separately and derived themes and subthemes from the data. After a process of comparing and discussing the data, consensus between the researchers was reached. Five themes and twelve subthemes emerged from thematic analysis of the transcripts (Table 3).

### 2.5. Ethical Considerations

Approval from the Ethics Committee of the Autonomous Community of Aragon was obtained prior to the beginning of the study (C.P.-C.I. PI20/097; 1 April 2020). Additionally, permission was sought from the participants’ employers prior to initiating the process of data collection. All participants voluntarily agreed to take part in the study procedures following an explanation of the study, which included information regarding their right to opt out at any time during the process and to decline to participate in the study without any effect on their professional career. All the participants were asked to give informed consent prior to being interviewed. Participant anonymity and confidentiality were guaranteed and safeguarded at all times, complying with the General Data Protection Regulation (RGPD 2016/679).

## 3. Results

The sociodemographic and cultural characteristics of our sample are presented in Table 4. The average age of the participants was 39. Nearly 90% of the participants were female. Regarding their marital status, 23% were single and just over three-quarters of the participants were either married or in a stable relationship. The vast majority of the participants defined themselves as having a European ethnic background (95%) and being middle class (94.9%). All of the participants had been born in the same country where they were working at the time of being interviewed. Almost 80% of our participants had not had any prior training in cultural competence, and approximately the same percentage stated that they did not belong to a culturally diverse family or group of friends. Finally, only a minority had lived or studied abroad for over three months (10.3%).

### 3.1. Theme 1. Relevance of Culture for Nursing

This theme integrates the nurses’ insights into what is cultural competence, their cultural constructions, and their experiences of working in a multicultural society.

#### 3.1.1. Subtheme 1.1. Defining Cultural Competence

The participants offered definitions of the concept of cultural competence and often linked it to the idea of adapting one’s practice to the patient’s needs.

*“Being culturally competent means trying to adapt to different cultures or taking into account what is important to other people, to the extent possible of course” (SN, Belgium).*

#### 3.1.2. Subtheme 1.2. Framing Cultural Care

Most of the participants had experience caring for patients from diverse cultural backgrounds. The SN and NM described their experiences in relation to specific life events and to the perceived similarities and differences between their own and other cultures.

*“It’s everything surrounding labour, childbirth, and death. These three are the parts that are most related to culture, the parts that are most different from us” (NM, Spain).*

*“I’ve realised over the years that immigrant people are just people too. If you deal with them in a human way, they offer normal concerns and you take certain customs into account” (NM, Belgium).*

### 3.2. Theme 2. Culture in the Healthcare Service

This theme describes the participants’ perception of how culture affects and shapes healthcare processes. The healthcare service was described as a cultural construction that influences healthcare, and which interacts with cultural constructions specific to cultural minority groups.

#### 3.2.1. Subtheme 2.1. Impact of Culture on Health and Healthcare

This subtheme includes the nurses’ perceptions of the impacts of culture on patient health. The participants also described their perceptions of how belonging to a minority group can affect healthcare and health outcomes.

*“There is dissatisfaction when patients do not receive care in accordance with their culture and traditions. This negatively affects the quality of care and health” (SN, Turkey).*

*“I don’t think their understanding of the information is different from yours, or mine, they understand it perfectly, but I think that we sometimes forget that their understanding of health and disease is culturally affected, and it sounds strange to them when you tell them exactly the same thing as you tell everyone else” (SN, Spain).*

#### 3.2.2. Subtheme 2.2. An Improper Use of the Healthcare System

The participants described what was perceived as the improper use of the health service by patients belonging to cultural minorities; they gave plenty of examples and often suggested that this inadequate use of the health service was rooted in their cultural background.

*“In their countries of origin, the healthcare services are a lot worse than ours … sometimes they come for nothing” (SN, Spain).*

*“Evidently, because it is a developed country where they can freely access the health service without any cost, well, of course they use the health service (A&E), even if their toe has been hurting for a month, because they know that in their countries there is no doctor, or it’s going to cost them an arm and a leg” (SN, Spain).*

In addition, they suggested that patients from cultural minority groups were either unprepared or unwilling to “follow the rules” imposed by the system. The SN often expressed that healthcare guidelines and norms were there to be followed by all. In fact, they remarked that no exceptions should be made for anyone, including for cultural reasons.

*“Sometimes these people do not comply with the rules” (SN, Portugal).*

*“I think that nurses’ ability to respond to the needs of people from other minorities is great but often people who turn to the health service are often not educated in the service that is provided to them” (SN, Portugal).*

#### 3.2.3. Subtheme 2.3. “Someone’s Gotta Give”—Patients versus Nurses

Despite the above, the participants acknowledged that both the healthcare staff and their culturally diverse patients needed to make an effort to adapt to one another, and pointed at “failings” on both sides.

*“Health professionals may not be prepared to deal with multiculturality. On the other hand, these citizens of different ethnic groups may not be adapted to our culture and to our health system” (SN, Turkey).*

Sometimes, the emphasis was put on the need to coexist in a multicultural environment.

*“We think that, in order to adapt, migrants should do as we do and that’s a mistake, no? Multiculturalism is about living with people from different cultures, not doing what the locals do because it’s them that have to adapt to us and not the other way around” (SN, Spain).*

Sometimes, the mission of the institution was described as the prevailing element.

*“Although each of us can have our own culture and values, we all have to understand the mission of the institution” (NM, Portugal).*

However, it was not infrequent for the participants to describe instances where they adapted their practice in order to meet their patients’ needs, even if sometimes they did so rather reluctantly:

*“In the end, we accept the wishes of the patient and the family” (NM, Belgium).*

*“Sometimes there is positive discrimination, you say ‘oh man, just don’t me any grief, you can all three come in’. And you see everyone else, for example, a patient who would love to have their son and their wife and their other son with them and they can’t, because we don’t let them, because there are rules, but that one … gypsies specifically, you let them do as they please to avoid arguing with them, it’s like ‘enough, I can’t take this anymore’” (NM, Spain).*

#### 3.2.4. Subtheme 2.4. An Individual Effort or an Organizational Enterprise

The participants described (generally) individual initiatives to address inequity in healthcare. Occasionally, the SN demanded more support from health management and from health decision and policy makers.

*“It’s hard to know everything about their lifestyle and their beliefs, but you have to adapt, you have to adapt and ask, as to the extent possible, because you can’t know everything” (NM, Spain).*

*“It may be easier for you (addresses another SN), but I am going to say something really negative here; I think the system is not prepared” (SN, Spain).*

### 3.3. Theme 3. Qualities of the Healthcare Professionals

This theme integrates the participants’ self-assessment of cultural competence, as well as the qualities or professional values that were described as contributing to a culturally competent nursing practice.

#### 3.3.1. Subtheme 3.1. Self-Assessment of Cultural Competence

Almost every interviewee said that they did not consider themselves culturally competent. Whereas some of them did feel comfortable with their perceived level of cultural competence, most of them considered that they still had much to learn. Some of the nurses emphasised their lack of knowledge about, and understanding of, specific minority cultures, whereas others suggested that a lack of cultural knowledge could be overcome by showing an empathic, respectful, and open attitude towards those who were different, and by confronting these situations with a predisposition to learn and adapt one’s practice.

*“I don’t think I am fully competent. Although I am culturally sensitive but sometimes, I am not able to get the root of patient’s health problem” (SN, Turkey).*

*“I feel confident and persevering in caring for people regardless of their culture” (SN, Portugal).*

Some professionals highlighted that professional experience or seniority in healthcare were determinant in improving one’s level of cultural competence.

*“I think I lack knowledge, loads. I make up for that with skill, the skill that you acquire after 39 years of service” (NM, Spain).*

*“I have the idea that younger colleagues have enormous difficulty in accepting the difference. That’s something that scares me. It is necessary to respect the difference, not to issue judgments” (NM, Portugal).*

#### 3.3.2. Subtheme 3.2. Predisposition to Culturally Competent Nursing Care

The participants acknowledged that professional experience was not the only factor influencing cultural competence. They suggested that nurses had to be predisposed and had to have a degree of cultural sensitivity in order to become culturally competent professionals. Other influencing factors included the quality of education or upbringing, certain values such as tolerance, and a passion for nursing.

*“My culture is related to the education I had; being understanding and empathetic, trying to understand the “other”, has a lot of influence on what I do on a day-to-day basis” (SN, Portugal).*

*“As a society (Turkish), we are a supportive, tolerant, and respectful society” (SN, Turkey).*

#### 3.3.3. Subtheme 3.3. Professional Values

The nurses made reference to a number of professional values contributing or leading to the provision of culturally competent nursing care. Interestingly, one of them was assertiveness. The participants suggested that assertiveness was necessary to provide culturally congruent care without completely abandoning one’s cultural principles.

*“I had to have a more assertive speech” (SN, Portugal).*

*“African moms can sometimes forget that you were going to make a home visit … but I am going to wait half an hour for them. There are limitations in everything, but I certainly do my best” (SN, Belgium).*

Another key professional value was empathy.

*“I believed that younger nurses need to exercise more empathy for citizens of different backgrounds” (SN, Belgium).*

*“I try to observe their gestures, their facial expressions, that gives you so much information, in truth because when you say that you don’t like something, it shows, or you say something that’s contrary to what they think, or whatever their culture dictates” (NM, Spain).*

Being open-minded was also cited as an essential requirement in order to become a culturally competent nurse. In fact, being open-minded was described as essential for individualised and holistic care.

*“Some people are open to it; you often have to try and push harder on that front if necessary. We may need to take more time to learn about other cultures” (SN, Belgium).*

*“I think so. That´s also a quality you have to possess as a midwife. You have to be soft as a midwife and be open to such things. We also ventilate of course; we don´t agree with everything but you have to be able to deal with it” (SN, Belgium).*

### 3.4. Theme 4. Challenges to Culturally Competent Care

Three main barriers or challenges to culturally competent care were described by the participants, namely language and communication, religion, and prejudices and stereotyping.

#### 3.4.1. Subtheme 4.1. Language and Communication Barriers

This subtheme describes the communication difficulties experienced by the SF and NM whilst caring for patients from minority cultural backgrounds. Generally, language was perceived as the most significant barrier when caring for diverse patients and their families.

*“Language is particularly one of the major obstacles to care and planning” (SF, Portugal).*

*“The biggest problem is communication” (SN, Turkey).*

The nurses reflected on the impacts of these barriers on the quality of patient care and the nurse–patient relationship.

*“We already have documentation in many languages, but sometimes we miss things in certain languages” (NM, Belgium).*

*“There are translators and there is also an effort within the hospital. If there is an urgent situation, I feel that there is definitely an effort to still understand each other” (SN, Belgium).*

In addition, they perceived that it was necessary to make an extra effort to breach the language barrier:

*“I think my attitude is definitely in order. Therefore, I am equally sorry that I cannot express myself well in all languages … we try to communicate at such times using pictograms” (SN, Belgium).*

*“We developed sign language … we use our body language actively” (SN, Turkey).*

However, language was not the only communication barrier observed and experienced by the nurses. Sometimes, the healthcare staff’s attitude when caring for people from a different cultural background was cited as a source of difficulty too.

*“In adult care I have had the feeling that the migrant population I don’t know … some staff, usually nurses, don’t waste time explaining certain things because they assume that they’ll never be understood” (SN, Spain).*

The participants described various efforts to address the language barrier; some professionals described it as an interesting challenge and others as the main obstacle for not providing culturally competent care. Some participants suggested that patients should also make an effort to overcome the language barrier.

*“It takes loads of time, to explain things well, to adapt, often due to our workload we don’t have that kind of time and dedication to help them access … Often also we don’t adapt to their circumstances, we speak fast … they say they have, but in truth they haven’t understood a thing” (SN, Spain).*

*“You can’t do more than your best. When the language barrier is high, it becomes difficult to provide appropriate care. We often get the feeling that we make a lot of effort, but the patient doesn’t always make the same effort in return” (SN, Belgium).*

Accompanying children often spoke the local language better than their adult relatives, and often they were charged with the responsibility of interpreting for their families.

*“Children often speak good Spanish and so they translate for their parents, and sometimes I have wondered, we are simply not aware of the burden and responsibility that we are dropping on these children’s littler shoulders” (SN, Spain).*

*“I find it shocking to have to use a child as an interpreter” (NM, Spain).*

#### 3.4.2. Subtheme 4.2. Religion and Spirituality

Religion was cited by the participants as a challenge to culturally competent care; they found it very hard to deal with situations that were religiously or spiritually significant.

*“Insecure … fearful of screwing up big time … or unknowingly disrespecting them, that scares me” (SN, Spain).*

*“I wouldn´t approach anything (religiously) differently because you always have certain prejudices. I did notice that my views on certain groups have changed” (NM, Belgium).*

#### 3.4.3. Subtheme 4.3. Prejudice and Stereotype in Healthcare

Occasionally, the participants stereotyped minority patients and tried to classify them according to what were perceived as specific culturally determined needs or traits, such as social problems including poverty and antisocial behaviour.

*“With few resources yes, if they come from a European country, like England, then of course not. You know, regardless of anything else, there are social problems, whatever it is. It is a given, no need to ask, no need to know anything else, there are going to be social problems” (SN, Spain).*

*“If someone came into the emergency room who had a dark skin colour, people would be more likely to assume that something was going to happen” (NM, Belgium).*

Protocols and physical spaces were described as promoting stereotyping and favouring a specific type of healthcare service. Specifically, some participants said that there was no space for patients to freely and intimately express themselves in a way that was congruent with their cultural tradition and values.

*“We are always talking about language difficulties with diverse patients, but I think there is an even bigger problem than that, even if we speak the same language, and that’s the open spaces in A&E and in every hospital. We need to redesign these spaces so that people can express their feelings” (SN, Spain).*

### 3.5. Theme 5. Becoming a Culturally Competeny Nurse

This theme describes the professionals’ experience and perception of training in cultural competence, which was identified and described as the root of a significant portion of the problems and difficulties described.

*“We do not have prior preparation to deal with people from other cultures, but in practice we are obliged to have it” (SN, Portugal).*

*“I miss cultural training … Sometimes I ask questions, or give them choices, I don’t know if I’m doing the right thing according to their culture” (NM, Spain).*

*“Our education is informal” (SN, Portugal).*

The participants expressed a need to receive formal training in cultural competence. Many of them said that cultural content was “casually” integrated into other courses and modules, and they described these learning experiences as insufficient. Some said that they searched for scientific articles on the topic. In addition, some of the participants mentioned that cultural encounters, occurring either in a professional or a personal context, were helpful yet insufficient for someone to become a culturally competent nursing professional.

*“It’s been an experience-based learning (…) Formal learning helps demystify pre-made ideas” (SN, Portugal).*

*“I learned by experience…I read articles and research” (SN, Turkey).*

Some of the participants suggested that cultural content should be integrated in the nursing curricula, whilst others thought that cultural content should be taught from primary education onwards.

*“From the moment you start your nursing studies. I mean, they should make us see that, that we are going to have to care for very different people, from different cultures, and simply that even if it was just brushstrokes, but they should expand our view” (SN, Spain).*

*“Education about ethnic origins should be provided in schools” (SN, Turkey).*

Finally, the participants highlighted that having an “etic” knowledge of their patients’ culture; that is, learning about their culture as something external and unconnected to them was insufficient to provide culturally competent care. Instead, they suggested that nurses should strive to grasp the other’s culture; that is, acquire an emic vision of their patients’ culture, where it is necessary to “experience the other’s culture” in order to achieve a better understanding of it.

*“It´s different because then you don´t experience the situation yourself. It could be efficient if a family from another culture tells their side of the story so that nurses can gain insight into their way of thinking” (NM, Belgium).*

*“When we have a patient, we have to involve the family and be open to them as well. If you do that, you are going to learn a lot about other cultures. But how do you teach someone to be open?” (NM, Belgium).*

## 4. Discussion

This article analysed the perceptions and experiences of cultural competence and care among 39 Portuguese, Turkish, Belgian and Spanish SN and NM.

Cultural competence is a complex construct that encompasses a number of related concepts, which include cultural desire, awareness, sensitivity, humility and safety, among others [5,11,17,18]. As mentioned in previous studies [19,20], the participants offered a variety of definitions of cultural competence. Their views on cultural competence revealed their cultural constructions and offered insight into their understanding of nursing in a multicultural society. Specifically, our participants made reference to the need to adapt one’s practice, highlighted some similarities and differences between their own and other minority cultures and identified specific life events, such as childbirth and death, when cultural differences were perceived as being more important. These topics are recurrent in the literature. Whereas health and disease, childbirth and death are common threads among all cultures and may be viewed as universal experiences, each individual’s experiences are different and unique, and are determined by their culture [21,22]. Thus, it is not surprising that the participants identified both childbirth and end of life as being culturally significant.

Further, the health service itself was described as a cultural construct, which circumscribed the way healthcare was delivered. We drew from Bibeau and Corin’s [23] definition of culture as a system of meaning, knowledge and action to explain the health service as a cultural construct. As a system of meaning, the culture of the health service helps individuals organise the multiple components of their small world into a coherent whole; as a system of knowledge, the health service sanctions normative behaviours and other acceptable ways of acting; as a system of action, the health service is a dynamic construct that allows its members to act upon their world so that new meanings, and thus new ways of acting, can be negotiated [24]. Thus, according to this model, the health service provides a platform for nurses to interpret their world and behave in a way that is expected and acceptable. When patients enter this world, their own culture interacts with not only the individual culture of the nurse, but also the health service as a cultural construct. In the course of this interaction, difficulties may arise that need to be addressed in order to establish an adequate nurse–patient relationship [25]. One way to address these difficulties is to engage in a dialogue to negotiate a different way of acting for either the patient or the nurse or both. In order to achieve this, good communication is critical for patients and nurses to feel comfortable in exchanging views, negotiating decisions and fully understanding the other party’s position and preferences [26]. However, there are a number of factors that may hinder this process.

Although as a system of action the health service is a dynamic entity [24], introducing new ways of acting can be complex and time-consuming. The SN described how often initiatives to address health inequities arose not from an organisation-wide strategy but through individual enterprises. In fact, the participants demanded more support from health management and from health decision and policy makers. This is in line with the findings from a recent systematic review [27] about the impacts of culturally responsive self-management interventions on health outcomes for minority populations, which proposed that rather than focusing on individual initiatives, it may be more effective to create empathic and responsive systems to improve the health of minority groups. However, the participants’ observations of the health service’s unpreparedness to provide equitable care for all somewhat clashed with their description of what, in their view, were examples of misuse of the health service by patients from diverse cultural backgrounds. Generally, these episodes were described in relation to the patients’ cultural background and the cultural constructions associated to their countries of origin and ethnicity. Specifically, the SN made reference to specific rules and regulations that should always be followed, without exception for cultural or other reasons. Interestingly, the NM considered that more often than not it should be the health service that tries to adapt to the needs of these patients in these cases, and not the other way around. In a recent systematic review, Curtis et al. [28] suggested that individual-level positionings for cultural competency are fundamentally limited in their ability to impact health inequities, and added that health services and organizations should be responsible for promoting equity through the structure of the healthcare environment, including factors such as accountability for equity, workplace stressors and diversity in the workforce and governance, among others. For example, our SN often cited time and work pressures, both stemming from the health service, as barriers to equitable, culturally competent care and worse health outcomes [29]. This is in agreement with previous authors [30] that emphasised the role of the health service in the creation of culturally safe and equitable environments.

Our participants explained and discussed their experience of working with patients from culturally diverse backgrounds. Most of the nurses referred to these encounters as positive and enriching. However, as discussed in previous studies [31], the participants’ discourse was not entirely free of a tendency to ethnocentrism. Our participants occasionally described their own culture as the norm and the expected way of being and behaving. Interestingly, the nurse managers’ discourse seemed to be slightly more “tolerant and open minded” than that of the staff nurses. In fact, some of them described themselves as mediators when conflict arose between the healthcare personnel and patients from cultural minority groups. Ethnocentrism has been cited in the nursing literature since before the 1980s as a barrier to effective care [32]. In contrast, the concept of cultural humility emerged as a way to help individuals identify their own biases and prejudices towards cultures different from one’s own, whilst taking responsibility for one’s actions and interactions with others, developing respectful partnerships with each patient by means of patient-centred care and acknowledging that it is impossible to be adequately knowledgeable about cultures other than one’s own [33]. Our participants expressed a lack of knowledge and skills to care for diverse patient groups in a sensitive manner. Sometimes they were aware of stereotyping when interacting with patients from cultures different from their own, yet they found it difficult to overcome this challenge.

Generally, language was perceived as the most significant barrier in the care process. Previous investigations have described suboptimal nursing care [34] for patients from diverse cultural backgrounds, as well as problems in the establishment of the nurse–patient relationship [35] when the language barrier was not addressed. Interestingly, medical interpreters were cited as useful resources but were often unavailable [36]. Instead, the nurses said that they used alternative strategies including pictograms and relatives, even children, as interpreters. Using means of communication other than professional interpreters to breach the language barrier is not uncommon [37]. However, as demonstrated by Flores et al. [38], the use of ad hoc interpreters or no interpreters at all may result in a higher likelihood of therapeutic errors.

Language was not the only barrier to effective communication between nursing staff and patients from minority backgrounds. For example, prejudice against specific groups was cited as one such barrier, namely prejudice against Gypsy Roma in Spain, Muslims in Belgium and Syrian refugees in Turkey. Wilson et al. [25] analysed the experiences of health inequity in New Zealand Māori and Gypsy Roma Travelers, and concluded that a lack of understanding and valuing of alternative worldviews contributes to poorer health outcomes in marginalised communities. Another recent study [39] carried out in France revealed that women, immigrants from Africa or overseas and Muslims were more likely to be discriminated against within healthcare. As nurses, it is important to find ways of ensuring culturally responsive, mindful and safe healthcare environments that respect and value alternative worldviews [25]. Our participants also mentioned that they felt out of their depth when dealing with religious differences and attending to patients’ spiritual needs. Religion and spirituality are important to a significant proportion of patients seeking care. However, religious beliefs are not always taken into account [40]. Regardless of their own religious background, it is important for nurses to recognise and accommodate their patients’ religious and spiritual needs, and to understand that patients and relatives may turn to their religious and spiritual beliefs when making decisions. Thus, health professionals should provide an opportunity for patients to discuss their religious and spiritual beliefs and tailor their evaluation and treatment to meet their specific needs [41].

The nurses’ self-perceptions of cultural competence ranged from inadequate to sufficient. This is in agreement with a recent study [20] analysing the level of cultural competence in a sample of undergraduates, recent graduates, registered nurses and clinical mentors. Some of the participants suggested that cultural competence can be learnt and acquired through professional experience, whereas others implied that a certain predisposition or cultural desire [18] was necessary to be able to provide culturally competent care. It has been suggested in the literature [9,42] that only through practice and reflection can cultural skills be refined, resulting in cultural competence. This appreciation coincides with Campinha-Bacote [18] and other theorists who described cultural competence as a growth process rather than a static evaluation performance outcome. In addition, the participants identified specific attitudes and values that were perceived as important to deliver culturally congruent nursing care to all, including empathy [43], assertiveness [44] and active listening [45]. Other factors identified by the participants as influencing their cultural competence were their education and upbringing, an intrinsic motivation to help others [46] and previous formal training in cultural competence. Across all four participating countries, the nurses suggested that cultural content should be integrated in the nursing curricula [47].

### Limitations

Whereas it was extremely interesting to analyse the perceptions and experiences of nurses who belong to the cultural majority within their respective societies, it would be interesting to include nurses from minority cultural backgrounds in future studies addressing these issues. Also, although we analysed the nurses’ testimonies as a whole, we acknowledge that specific differences exist in the way the nurses perceive, experience and learn about cultural competence. These differences emerge from the educational and healthcare systems and the cultures and societies represented in each country, and must be taken into account when addressing these issues in practice. We also wish to highlight the fact that both nurses and midwives took part in this investigation, owing to the fact that midwifery is a nursing specialisation in two of the countries involved in this investigation. Finally, it is important to note that as pointed out in the methods section, there was inconsistency in the methodological approaches used to collect data. Specifically, focus groups were used in Spain, Portugal and Tukey to collect information, whilst personal interviews were carried out in Belgium, meaning it is not possible to guarantee the solidity of the results.

## 5. Conclusions

Our findings reinforce the notion that European health services are generally unprepared to deliver safe, equitable, culturally congruent care to all. Cultural competence is linked to the idea of adapting one’s practice to the needs of patients from diverse cultural backgrounds. However, there are challenges that limit these changes to nursing practice, namely language and communication difficulties, prejudice and stereotype in the health service, a tendency for ethnocentrism, a lack of education and training in cultural competence and a lack of support from the health service to facilitate new ways of acting.

## Figures and Tables

**Table 1:** Topic guide for discussion groups with qualified nurses.

Opening Question
We are interested in hearing about your experience of working with patients from diverse cultural backgrounds. Before we begin, can you tell us what the term cultural competence means to you?
**Follow-Up Questions**
How do you think cultural differences affect health (i.e., ethnicity, nationality, religion, etc.)? What impacts do you think being from a diverse cultural background has on patients/families/groups? Do you think the needs of patients belonging to minority groups are being met currently in healthcare services? Could you explain which of them are attended to and which are not? Why do you think this happens? Have you ever considered how your own culture influences your nursing practice? Do you think qualified nurses and other healthcare professionals are sufficiently prepared to deliver culturally appropriate care to patients from diverse cultural backgrounds? Are you confident in your level of cultural competence to look after patients from diverse cultural backgrounds? How did you gain the knowledge/skills to look after patients from diverse cultural backgrounds? Have you had any formal training and/or experiential learning in cultural competence? What did it consist of? Was it useful? Regarding your experiences of working with multicultural patients, please can you tell us about any especially significant experiences you have had to date? Do you remember how you felt in that situation and what you learnt from that experience? How do you deal with difficulties/conflicts emerging from working with patients from different cultural backgrounds? Can you think of any recent examples? What do you think are your needs in the way of education/training in cultural competence?

**Table 2:** Topic guide for semi-structured interviews with NM.

Opening Question
I am interested in hearing about your experience of working with patients from diverse cultural backgrounds. Please can you tell us about any experiences that you have had to date?
**Follow-Up Questions**
What do you understand transcultural nursing care to mean? Would you say that transcultural nursing care is needed in the service that you lead/manage? Do you think that health disparities and/or inequalities exist between patients belonging to minority groups and the large population majority? Can you give any examples? What, in your opinion, are the challenges of working with patients who are from diverse cultural backgrounds? How do you cope with these challenges in your policy/strategic management? Are there any human/material resources at your disposal to help provide culturally safe and appropriate care to patients from diverse cultural backgrounds (i.e., interpreting services, religious services, etc.)? Can you describe them? Do they get used? What aspects of the healthcare service would you like to see changed to improve health outcomes for patients from diverse cultural backgrounds? What aspects relating to caring for patients from diverse cultural backgrounds do you think should be taught to nurses? Have you ever considered how your own culture influences your leadership? Are you confident in your level of cultural competence to lead/help other nurses deliver culturally appropriate care for diverse patient populations? Have you had any formal training and/or experiential learning in cultural competence? What do you think are your training needs in this area? What are the training needs of the nursing workforce? What are the training needs of the management staff? How do you deal with conflict emerging from working with patients from diverse cultural backgrounds?

**Table 3:** Themes and subthemes.

Themes	Subthemes
1. Relevance of culture for nursing	Defining cultural competence
	Framing cultural care
2. Culture in the healthcare service	Impact of culture on health and healthcare
	Improper use of the healthcare system
	“Someone’s gotta give”—patients versus nurses
	An individual effort or an organisational enterprise
3. Qualities of the healthcare professionals	Self-assessment of cultural competence
	Predisposition to culturally competent nursing care
	Professional values
4. Challenges to culturally competent care	Language and communication barriers
	Religion and spirituality
	Prejudice and stereotypes in healthcare
5. Becoming a culturally competent nurse	

**Table 4:** Sociodemographic and cultural characteristics of the sample (n = 39).

	Manager (n = 11)	Nurse (n = 28)	Total (n = 39)
	Mean (SD)	Mean (SD)	Mean (SD)
Age (years)	42.44 (7.35)	37.45 (8.60)	38.90 (8.45)
Clinical work experience (years)	20.89 (6.73)	15.96 (8.06)	17.23 (7.95)
	Freq (%)	Freq (%)	Freq (%)
Gender			
Male	1 (9.1%)	4 (14.2%)	5 (12.7%)
Female	10 (90.9%)	24 (85.8%)	34 (87.3%)
Marital status			
Married	11 (100%)	17 (60.7%)	28 (71.8%)
Partner	0 (0%)	2 (7.2%)	2 (5.2%)
Single	0 (0%)	9 (32.1%)	9 (23%)
Geographical cluster			
Asian	0 (0%)	1 (3.6%)	1 (5%)
European	11 (100%)	27 (96.4%)	38 (95%)
Religious affiliation			
Atheism	0 (0%)	1 (3.6%)	1 (2.6%)
Catholicism	8 (72.7%)	15 (53.6%)	23 (59%)
Islam	3(27.3%)	4 (14.3%)	7 (17.9%)
None	0 (0%)	8 (28.5%)	8 (20.5%)
Adherence to religion			
Non-practicing	3 (27.3%)	14 (50%)	17 (40%)
Practicing	8 (72.7%)	14 (50%)	22 (60%)
Socioeconomic level			
High social class	2 (18.2%)	0 (0%)	2 (5.1%)
Middle social class	9 (81.8%)	28 (100%)	37 (94.9%)
Country of birth			
Belgium	3 (27.3%)	4 (14.3%)	7 (18%)
Portugal	2 (18.1%)	6 (21.4%)	8 (20.5%)
Spain	3 (27.3%)	13 (46.4%)	16 (41%)
Turkey	3 (27.3%)	5 (17.9%)	8 (20.5%)
Country of work			
Belgium	3 (27.3%)	4 (14.3%)	7 (18%)
Portugal	2 (18.1%)	6 (21.4%)	8 (20.5%)
Spain	3 (27.3%)	13 (46.4%)	16 (41%)
Turkey	3 (27.3%)	5 (17.9%)	8 (20.5%)
Mother tongue			
Dutch	3 (27.3%)	4 (14.3%)	7 (18%)
Portuguese	2 (18.1%)	6 (21.4%)	8 (20.5%)
Spanish	3(27.3%)	13 (46.4%)	16 (41%)
Turkey	3 (27.3%)	5 (17.9%)	8 (20.5%)
Other languages			
No	3 (27.3%)	10 (35.7%)	13 (33.3%)
Yes	8 (72.7%)	18 (64.3%)	26 (66.7%)
Belonging to a culturally diverse family			
No	8 (72.7%)	24 (85.7%)	32 (82.1%)
Yes	3 (27.3%)	4 (14.3%)	7 (17.9%)
Prior cultural competence training			
No	8 (72.7%)	23 (82.1%)	31 (79.5%)
Yes	3 (27.3%)	5 (17.9%)	8 (20.5%)
Prior/current voluntary work with patients from diverse cultural backgrounds			
No	9 (81.8%)	20 (71.4%)	29 (74.4%)
Yes	2 (18.2%)	8 (28.6%)	10 (25.6%)
Experience in caring for patients from diverse cultural backgrounds			
No	3 (27.3%)	17 (60.7%)	20 (51.3%)
Yes	8 (72.7%)	11 (39.3%)	19 (48.7%)
Lived/studied abroad for at least 3 months			
No	11 (100%)	24 (85.7%)	35 (89.7%)
Yes	0 (0%)	4 (14.3%)	4 (10.3%)

## Data Availability

The data presented in this study are available on request from the corresponding author. The data are not publicly available due to ethical restrictions.

## Supplementary Materials

The following supporting information can be downloaded at: https://www.mdpi.com/article/10.3390/nursrep12020034/s1. Table S1: Examples of representative quotes from each theme and subtheme.
