# Supplementary material for: Qualified Nurses’ Perceptions of Cultural Competence and Experiences of Caring for Culturally Diverse Patients: A Qualitative Study in Four European Countries
Source: Nurs Rep. 2022 May 5;12(2):348–64. doi: 10.3390/nursrep12020034 (PMC9149935; doi:10.3390/nursrep12020034)
Supplement: Supplementary file 1 [file nursrep-12-00034-s001.zip › nursrep-1652417-supplementary.pdf]

Table S1. Examples of representative quotes from each theme and subtheme.

**Theme**	**Subtheme**	**Quotes**
Relevance of culture for nursing	Defining cultural competence	*"Being culturally competent means trying to adapt to different cultures or taking into account what is important to other people, to the extent possible of course" (SN, Belgium)*<br>*"In my opinion, being culturally competent means having an understanding of different cultures and customs in order to better understand other cultures" (SN, Belgium)*<br>*"To understand that the other person has their own culture, their beliefs; he thinks that that's the best for him and, at the end of the day, it's about adapting to one another, that way there's no need for someone to have to change, there's no need to change someone's way of thinking, in the end" (SN, Spain)*
	Framing cultural care	*"It's everything surrounding labor, childbirth and death. These three are the parts that are most related to culture, the parts that are most different from us" (NM, Spain)*<br>*"I've realized over the years that immigrant people are just people too. If you deal with them in a human way, they offer normal concerns and you take certain customs into account" (NM, Belgium*)<br>*"Culture, social, educational, economic level, they all influence health, […] although implementing a community intervention to improve the nutritional habits of a sector of the population doesn't look as good as buying a new MR scan (smiles ironically) (SN, Spain)*<br>*"Culture affects how you are as a person and as a leader anyway" (NM, Belgium)*
Culture in the healthcare service	Impact of culture on health and health care	*"There is dissatisfaction when patients do not receive care in accordance with their culture and traditions. This negatively affects the quality of care and health" (SN, Turkey)*

		"The expectation that they will recover in a Belgian hospital is enormous. Patients often think that healthcare in Belgium is much better, but we can´t perform miracles either" (SN, Belgium)<br><br>*"It (culture) affects health negatively" (SN, Turkey)*<br><br>*"I don't think their understanding of the information is different from yours, or mine, they understand it perfectly, but I think that we sometimes forget that their understanding of health and disease is culturally affected, and it sounds strange to them when you tell them exactly the same thing as you tell everyone else (SN, Spain)*<br><br>*"There are plenty of difficulties for cultural reasons that we don't know how to address, nor them; that represents a huge problem when you see them in clinic, and also, they have loads of problems to access the health service" (SN, Spain)*<br><br>*"The means to care for these people adequately at a macro level are just insufficient" (SN, Spain)*<br><br>*"I think that there is some kind of inequity, but it's because, you know, it's because we just don't have enough time to stop and listen to them" (NM, Spain)*<br><br>*"Of course we need staff, to have time. Because time is everything. It is time to care I am talking about" (NM, Spain)*<br><br>*"It may be easier for you (addresses another SN), but I am going to say something really negative here; I think the system is zero prepared" (SN, Spain)*
	Stereotype and inequity in healthcare	*"With few resources yes, if they come from a European country, like England, then of course not. You know, regardless of anything else, there are social problems, whatever it is. It is a given, no need to ask, no need to know anything else, there are going to be social problems" (SN, Spain)*<br><br>*"If someone came into the emergency room who had a dark skin color, people would be more likely to assume that something was going to happen" (NM, Belgium)*

		"We are always talking about language difficulties with diverse patients, but I think there is an even bigger problem than that, even if we speak the same language, and that's the open spaces in A&E and in every hospital. We need to redesign these spaces so that people can express their feelings" (SN, Spain)<br>*"It was important then that we have a separated room for each religion to wash the people. During nursing, information is also given for each department on what to do and what no to do, because the ritual in case of death is very different in different religions"* (NM, Belgium)
	An improper use or an improper service	*"In their countries of origin, the healthcare services are a lot worse than ours…, sometimes they come for nothing"* (SN, Spain)<br>*"There is inequality, not because they don't receive… […] It is likely that they don't know what services they have access to. Some of them are clever, we all know who we are talking about, and then some others are super legal, and that people are always missing out of aid and information"* (NM, Spain)<br>*"Evidently, because it is a developed country where they can freely access the health service without any cost, well, of course they use the health service [A&E], even if their toe has been hurting for a month, because they know that in their countries there is no doctor, or it's going to cost them an arm and a leg"* (SN, Spain)<br>*"The first contact [with the health service] is always demanding, there is suspicion and mistrust"* (SN, Spain)<br>*"Sometimes these people do not comply with the rules"* (SN, Portugal)<br>*"My competence in patient care is more efficient if they know their rights and duties"* (SN, Portugal)<br>*"I think that nurses 'ability to respond to the needs of people from other minorities is great but often people who turn to the health service are often not educated for the service that is provided to them"* (SN, Portugal)

		"At hospital strategic level…, I don't know what problems, I suppose that they probably observed that there were too many problems with that type of culture, because it's true, we always end up arguing, because they couldn't understand our situation as professionals working in an A&E service, and then we weren't able to understand their beliefs" (NM, Spain).
	"~~Someone's~~ Something's gotta give": Patients versus healthcare professionals	*"We think that, in order to adapt, migrants should do as we do and that's a mistake, no? Multiculturalism is about living with people from different cultures, not doing what the locals do because it's them that have to adapt to us and not the other way around" (SN, Spain)*<br><br>*"Bad, you know? […] Because these things are important and necessary and you find yourself there, facing a barrier that is very hard, then you lose so much time. In my ward, for example, we are always running behind and it's like saying: 'come on, that's all we needed!'" (SN, Spain)*<br><br>*"During my first year as a nurse in the hospital, that must have been less the case because I was pulled along by culture of the ward. You have to find your place in that specific culture. Now, as head nurse, I try to take everyone´s views on board, both those of residents and their families and those of the staff" (NM, Belgium)*<br><br>*"It's hard to know everything about their lifestyle and their beliefs, but you have to adapt, you have to adapt and ask, as to the extent possible, because you can't know everything" (NM, Spain)*<br><br>*"Sometimes there is positive discrimination, you say: 'oh man, just don't me any grief, you can all three come in'. And you see everyone else, for example, a patient who would love to have their son and their wife and their other son with them and they can't, because we don't let them, because there are rules, but that one…, Gypsies specifically, you let them do as they please to avoid arguing with them, it's like: 'enough, I can't take this anymore'" (NM, Spain)*<br><br>*"In the end, we accept the wishes of the patient and the family" (NM, Belgium)*

		"Although each of us can have its own culture and values, we all have to understand the mission of the institution" (NM, Portugal)<br>*"Health professionals may not be prepared to deal with multiculturality. On the other hand, these citizens of different ethnic groups may not be adapted to our culture and to our health system" (SN, Turkey)*
Qualities of the healthcare professionals	Self-assessment of cultural competence	*"I don't think I am fully competent. Although I am culturally sensitive but sometimes, I am not able to get the root of patient´s health problem" (SN, Turkey)*<br>*"We can only look at superficial problems and may not be able to understand in-depth problems. I do not think I am competent enough" (SN, Turkey)*<br>*"I feel confident and persevering in caring for people regardless of their culture" (SN, Portugal)*<br>*"I want to think so, that we are well prepared or that we are getting better at it [being culturally competent]" (SN, Spain)*<br>*"I think I lack knowledge, loads. I make up for that with skill, the skill that you acquire after 39 years of service" (NM, Spain)*<br>*"I have enough confidence in that, but wisdom comes with years so I can certainly still learn a lot" (NM, Belgium)*<br>*"I have the idea that younger colleagues have an enormous difficult in accepting the difference. That´s something that scares me. It is necessary to respect the difference, not to issue judgments" (NM, Portugal)*
	Predisposition to culturally competent nursing care	*"My culture is related to the education I had: being understanding and empathetic, trying to understand the "other", has a lot of influence on what I do on a day-to-day basis" (SN, Portugal)*<br>*"As a society [Turkish], we are supportive, tolerant and respectful society" (SN, Turkey)*

		"I am a nurse, but really, I mean, my nursing practice is what I have learnt from other nurses, it's during placement when I've learnt the most from other people who have the same culture as me, and they are the ones who taught me how to be a nurse" (SN, Spain)<br>*"We as Portuguese are a people that adapts easily. As nurses we adapt doubly" (SN, Portugal)*
	Professional values	*"From our perspective, I feel that you choose healthcare, you put your principles aside. If nurses/midwives have a certain opinion about something we are going to discuss it among colleagues but not express, it harshly towards patients" (SN, Belgium)*<br>*"I had to have a more assertive speech" (SN, Portugal)*<br>*"African's moms can sometimes forget that you were going to make a home visit (…), but I am going to wait half an hour for them. There are limitations in everything, but I certainly do my best" (SN, Belgium)*<br>*"I believed that younger nurses need to exercise more empathy for citizens of different backgrounds" (SN, Belgium)*<br>*"I have been doing this work for 27 years and I still do it every day with heart and soul. People are also very grateful. People from some cultures tend to give hands all the time" (SN, Belgium)*<br>*"[…] I try to observe their gestures, they facial expression, that gives you so much information, in truth, because when you say that you don't like something, it shows, or you say something that's contrary to what they think, or whatever their culture dictates…" (NM, Spain)*<br>*"Some people are open to it; you often have to try and push harder on that front if necessary. We may need to take more time to learn about other cultures" (SN, Belgium)*<br>*"I think so. That´s also a quality you have to possess as a midwife. You have to be soft as a midwife and be open to such things. We also ventilate of course; we don´t agree with everything but you have to be able to deal with it" (SN, Belgium)*

Challenges to culturally competent care	Language and communication barriers	*"Language is particularly one of the major obstacles to care and planning" (SF, Portugal)*<br>*"The biggest problem is communication" (SN, Turkey)*<br>*"We already have documentation in many languages, but sometimes we miss things in certain languages" (NM, Belgium)*<br>*"There are translators and there is also an effort within the hospital. If there is an urgent situation, I feel that there is definitely an effort to still understand each other" (SN, Belgium)*<br>*"I think my attitude is definitely in order. Therefore, I am equally sorry that I cannot express myself well in all languages […] we try to communicate at such times using pictograms" (SN, Belgium)*<br>*"We developed the sign language [….], we use our body language actively" (SN, Turkey)*<br>*"In adult care I have had the feeling that the migrant population I don't know… Some staff, usually nurses, don't waste time explaining certain things because they assume that they'll never be understood" (SN, Spain)*<br>*"We try to get pictograms through occupational therapists. We also try to get interpreters, but it often takes them a long time to get there" (SN, Belgium)*<br>*"It takes loads of time, to explain things well, to adapt, often due to our workload we don't have that kind of time and dedication to help them access… Often also we don't adapt to their circumstances, we speak fast…, they say they have, but in truth they haven't understood a thing" (SN, Spain)*<br>*"You can't do more than your best. When language barrier is high, it becomes difficult to provide appropriate care. We often get the feeling that we make a lot of effort, but the patient doesn't always make the same effort in return" (SN, Belgium)*<br>*"Children often speak good Spanish and so they translate for their parents and, sometimes, I have wondered, we are simply not aware of the burden and responsibility that we are dropping on these children's littler shoulders" (SN, Spain)*

		"I find it shocking to have to use a child as an interpreter" (NM, Spain)
	Other challenges	*"Insecure…, fearful of screwing up big time…, or unknowingly disrespecting them, that scares me" (SN, Spain)*<br>*"I fear any religion that is different from mine; I'm scared of screwing up" (SN, Spain)*<br>*"I wouldn´t approach anything [religiously] differently because you always have certain prejudices. I did notice that my views on certain groups have changed" (NM, Belgium)*<br>*"[…] you learn from what you encounter. You learn by falling and getting back up" (SN, Belgium)*
Becoming a culturally competent nurse		*"We do not have prior preparation to deal with people from other cultures, but in practice we are obliged to have it" (SN, Portugal)*<br>*"I miss cultural training… […] Sometimes I ask questions, or give them choices, I don't know if I'm doing the right thing according to their culture" (NM, Spain)*<br>*"We do not have basic preparation for this" (SN, Portugal)*<br>*"Our education is informal" (SN, Portugal)*<br>*"You learn a lot indeed doing it yourself and by asking questions, there are many people open about culture-related questions. I didn´t know about the Chinese culture, for example, but when I visit them at home, I get the chance to ask them questions about it" (SN, Belgium)*<br>*"I have been an experience-based learning (…) Formal learning helps demystify pre-made ideas" (SN, Portugal)*<br>*"I learned by experience…I read articles and research" (SN, Turkey)*<br>*"I am reading articles on this subject" (SN, Turkey.)*<br>*"I think that´s (education) done a lot. There is a lot of talk about different cultures. If there has been a case where a colleague didn´t know what to do it is discussed" (SN, Belgium)*

	"This should be taught at university, I don't know how to explain it, to learn how to face day-to-day situations with Muslims, or Chinese, like, look, when a Chinese baby is born, the parents don't want to hold him, whereas we do" (NM, Spain)<br>*"From the moment to start your nursing studies. I mean, they should make us see that, that we are going to have to care for very different people, from different cultures and simply that, even if it was just brush-strokes, but they should expand our view…" (SN, Spain)*<br>*"It should be integrated into undergraduate education" (SN, Turkey)*<br>*"Education about ethnic origins should be provided in schools" (SN, Turkey)*<br>*"The new generations of nurses have developed a better cultural background, and that creates stronger teams" (SN, Belgium)*<br>*"It´s different because then you don´t experience the situation yourself. It could be efficient if a family from another culture tells their side of the story so that nurses can gain insight into their way of thinking" (NM, Belgium)*<br>*"When we have a patient, we have to involve the family and be open to them as well. If you do that, you are going to learn a lot about other cultures. But how do you teach someone to be open?" (NM, Belgium)*
